# Base editing‐mediated perturbation of endogenous PKM1/2 splicing facilitates isoform‐specific functional analysis in vitro and in vivo

**DOI:** 10.1111/cpr.13096

**Published:** 2021-07-09

**Authors:** Jianxiang Lin, Susu Wu, Qingmei Shen, Jie Liu, Shisheng Huang, Guangdun Peng, Yunbo Qiao

**Affiliations:** ^1^ Precise Genome Engineering Center School of Life Sciences Guangzhou University Guangzhou China; ^2^ Centre for Cell Lineage and Atlas (CCLA), Bioland Laboratory (Guangzhou Regenerative Medicine and Health Guangdong Laboratory) Guangzhou China; ^3^ School of Life Science and Technology ShanghaiTech University Shanghai China

**Keywords:** base editing, *PKM*, RNA splicing, zebrafish

## Abstract

**Objectives:**

*PKM1* and *PKM2*, which are generated from the alternative splicing of *PKM* gene, play important roles in tumourigenesis and embryonic development as rate‐limiting enzymes in glycolytic pathway. However, because of the lack of appropriate techniques, the specific functions of the 2 *PKM* splicing isoforms have not been clarified endogenously yet.

**Materials and methods:**

In this study, we used CRISPR‐based base editors to perturbate the endogenous alternative splicing of *PKM* by introducing mutations into the splicing junction sites in HCT116 cells and zebrafish embryos. Sanger sequencing, agarose gel electrophoresis and targeted deep sequencing assays were utilized for identifying mutation efficiencies and detecting *PKM1/2* splicing isoforms. Cell proliferation assays and RNA‐seq analysis were performed to describe the effects of perturbation of *PKM1/2* splicing in tumour cell growth and zebrafish embryo development.

**Results:**

The splicing sites of *PKM*, a 5’ donor site of GT and a 3’ acceptor site of AG, were efficiently mutated by cytosine base editor (CBE; BE4max) and adenine base editor (ABE; ABEmax‐NG) with guide RNAs (gRNAs) targeting the splicing sites flanking exons 9 and 10 in HCT116 cells and/or zebrafish embryos. The mutations of the 5’ donor sites of GT flanking exons 9 or 10 into GC resulted in specific loss of *PKM1* or *PKM2* expression as well as the increase in *PKM2* or *PKM1* respectively. Specific loss of *PKM1* promoted cell proliferation of HCT116 cells and upregulated the expression of cell cycle regulators related to DNA replication and cell cycle phase transition. In contrast, specific loss of *PKM2* suppressed cell growth of HCT116 cells and resulted in growth retardation of zebrafish. Meanwhile, we found that mutation of *PKM1/2* splicing sites also perturbated the expression of non‐canonical *PKM* isoforms and produced some novel splicing isoforms.

**Conclusions:**

This work proved that CRISPR‐based base editing strategy can be used to disrupt the endogenous alternative splicing of genes of interest to study the function of specific splicing isoforms in vitro and in vivo. It also reminded us to notice some novel or undesirable splicing isoforms by targeting the splicing junction sites using base editors. In sum, we establish a platform to perturbate endogenous RNA splicing for functional investigation or genetic correction of abnormal splicing events in human diseases.

## Graphical abstract

Because of the lack of appropriate techniques, the specific functions of the two *PKM* splicing isoforms have not been clarified endogenously yet. In this study, we used CRISPR‐based base editors to perturbate the endogenous alternative splicing of *PKM* by introducing mutations into the splicing junction sites in HCT116 cells and zebrafish embryos. It's proved that CRISPR‐based base editing strategy can be used to disrupt the endogenous alternative splicing of genes of interest to study the function of specific splicing isoforms in vitro and in vivo

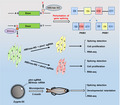

## 1 INTRODUCTION

RNA alternative splicing, which can splice one gene into multiple isoforms, is an important part of transcriptome modification, and different isoforms may have different functions in animal development, cell growth and disease.^1^, ^2^, ^3^ The conserved 5’ splice donor sites of GT and 3’ splice acceptor sites of AG, which are conserved in over 97% of transcripts as we previously demonstrated,^4^ are recognized in the first step of alternative splicing. The genetic mutations within the exon‐intron borders can change the splicing skipping rules and result in multiple genetic diseases.^5^ With the development of high‐throughput sequencing, more and more novel splicing isoforms have been discovered in embryo development^4^ and disease.^5^ In recent years, isoform‐specific functions have been extensively elucidated, and therapeutic targeting of splicing has been considered as an essential strategy for cancer therapy.^6^, ^7^ Therefore, precise interpretation of isoform‐specific function is of great importance.

Pyruvate kinase (PK), a rate‐limiting glycolytic enzyme, catalyses the irreversible transphosphorylation between phosphoenolpyruvate and adenosine diphosphate and produces pyruvate and ATP.^8^ In mammals, the PK family consists of 4 isoforms that are encoded by 2 genes, *PKLR* and pyruvate kinase muscle (*PKM*), with tissue‐specific features. *PKM1* and *PKM2*, which contain coding sequences encoded by exons 9 and 10, respectively, are produced by alternative splicing of the primary *PKM* RNA transcripts. *PKM1* is constitutively active and always expressed in terminally differentiated tissues, such as muscle and brain, whereas *PKM2* is expressed in proliferating tissues and tumour cells with anabolic functions.^9^, ^10^ *PKM2* that is upregulated in most cancer cells emerges as an attractive target for cancer therapy.^11^ Although the functions of *PKM* isoforms have been widely described by overexpression or knock‐in of isoform‐specific *PKM*,^11^, ^12^ specific depletion of *PKM1* or *PKM2* and modulation of alternative splicing of *PKM* gene in an endogenous context remain to be a great challenge for loss‐of‐function analysis.

Clustered regularly interspersed short palindromic repeats (CRISPR/Cas9) become the most important tool for functional analysis for its high efficiency and convenience.^13^ Based on CRISPR technology, cytosine base editors (CBEs) and adenine base editors (ABEs) have been developed for precisely introducing C‐to‐T or A‐to‐G conversions without double‐strand breaks.^14^, ^15^ By utilizing the advantages of base editing, base editors and CRISPR‐guided AID have applied to modulate RNA splicing by mutating the splice GT/AG sites.^16^, ^17^, ^18^ Therefore, we used the recently developed base editors, ABEmax‐NG^18^ and BE4max^19^ with optimized codons and expanded editing scope, to investigate the isoform‐specific functions of *PKM* in cultured cancer cells and zebrafish embryos. In the present study, we established in intro and in vivo models to successfully disrupt endogenous alternative splicing of *PKM* genes, and isoform‐specific functions and transcriptomes as well as unexpected splicing isoforms were depicted.

## 2 MATERIALS AND METHODS

### 2.1 Plasmid construction

ABEmax‐NG and BE4max were kind gifts from Dr Xingxu Huang's laboratory from ShanghaiTech University. Oligos used for sgRNA construction were synthesized, annealed and cloned into BsaI site of the pGL3‐U6‐sgRNA‐PGK‐puromycin (Addgene, 51133) expression vector with T4 DNA ligase (NEB). The sequences for synthesis are listed in Table S1.

### 2.2 Cell culture and transfection

HCT116 cell, a human colorectal cancer cell line, was originally obtained from ATCC. HCT116 cells were cultivated in Dulbecco's modified eagle medium (DMEM) with 10% foetal bovine serum (FBS) and 1% penicillin/streptomycin, and were incubated in 37°C with 5% CO_2_. Cells were seeded on 6‐well plates and transfected with ABEmax‐NG or BE4max and sgRNA plasmids using EZtrans (AC04L099; Shanghai Life iLab Biotech) according to the manufacturer's instructions. Puromycin (4 ug/ml; Merck, 540411) was supplemented after transfection for 24 h for screening cells expressing pGL3‐U6‐sgRNA‐PGK‐puromycin, and cells were collected at 72 h post‐transfection. For establishing single clones with expected mutations, transfected cells with high‐mutation efficiency were plated onto 96‐well plate with about 50 cells. After proliferation for 10 days, single and round clones were seeded onto 24‐well plate. Cells from these clones were subjected to DNA extraction and genotyping. Clones with expected mutations were passaged for subsequent experiments.

### 2.3 Genomic DNA extraction and genotyping

Cultured cells were rinsed with PBS and genomic DNA was isolated with QuickExtract™ DNA Extraction Solution (Lucigen) as we previously described.^20^ Genomic DNA from zebrafish (*Danio rerio*) embryos was extracted by using alkaline lysis method. DNA fragments containing the targeting sites were amplified by PCR with Phanta Max Super‐Fidelity DNA polymerase (Vazyme; P505), and then the amplified products were subjected to Sanger sequencing. The results from Sanger sequencing were uploaded to EditR (https://moriaritylab.shinyapps.io/editr_v10/) for calculating the mutation rates. The primers used for PCR are listed in Table S2.

### 2.4 Microinjection of zebrafish

Female zebrafish at latency period were ovulated and mated with males. Guide RNA (gRNA) (50 ng/ul) and BE4max (100 ng/ul) mRNA were mixed for microinjection. The mixtures were injected into the zygotes and the zygotes were cultivated in water with 0.001% methylthionine chloride at 5% of CO_2_ with 37°C. After cultured for 24 h, the tails from obtained zebrafish embryos were subjected to genotyping. The embryos with high mutation efficiency were cultivated for 3 months, and zebrafishes were subjected to targeted deep sequencing, phenotyping and RNA sequencing (RNA‐seq).

### 2.5 RT‐PCR and isoform detection

Total RNA from HCT116 cells and zebrafish tissues were extracted using Trizol reagents according to the manufacturer's protocol. Two micrograms of RNA was subjected to RNA‐seq, and 1 ug of RNA was reverse transcribed into cDNA by using PrimeScript RT Master Mix (Vazyme). The cDNA was amplified by PCR with Phanta Max Super‐Fidelity DNA Polymerase (Vazyme; P505) with isoform‐specific primers, and then the amplified products were analysed on 1% agarose gel. The primers used for PCR are listed in Table S3.

### 2.6 Cell proliferation assay

A total of 10,000 wild‐type and mutated (from single‐clone) HCT116 cells were plated onto 96‐well plate. Cell growth was measured with Cell Counting Kit‐8 (CCK‐8) (Vazyme, China) at 24, 48, 72 and 96 h. Three independent replicates were performed and presented.

### 2.7 Targeted deep sequencing

DNA fragments containing the on‐target sites were amplified from genomic DNA using Phanta Max SuperFidelity DNA Polymerase (Vazyme; P505). The paired‐end sequencing of PCR amplicons was conducted using Illumina Nextseq 500 (2 × 150) platform at Novogene, China. Analysis was performed as previously described.^21^ Briefly, BWA and Samtools were employed for mapping the pair‐end reads to human genome, and VarDict was used to call single‐nucleotide variants and insertions and deletions (indels) in amplicon aware mode with default parameters. Primers of targeted deep sequencing are listed in Table S4.

### 2.8 RNA‐seq and analysis

Total RNA extracted from HCT116 cells and zebrafish tissues was subjected to RNA‐seq using Illumina Nova‐seq platform at Novogene, China. RNA‐seq data analysis was performed as we previously described.^22^, ^23^ The paired‐end RNA‐Seq reads were mapped to the GRCh38/hg38 reference genome using STAR (v2.5.3a), and gene expression was quantified to FPKM for each gene using RSEM (v1.3.0). Raw read counts were estimated by featureCounts (v1.5.2), and DESeq2 (v1.22.1) was used for differential expression analysis. Heatmaps were generated using the FPKM values with pheatmap (v1.0.10). Functional annotation based on gene ontology (GO) and Kyoto Encyclopedia of Genes and Genomes (KEGG) pathway was performed using R/Bioconductor package clusterProfiler (v3.10.0). The genome browser snapshot was shown by IGV viewer to present the exon distributions of transcripts.

### 2.9 In vitro transcription and animal experiment in zebrafish

T7‐sgRNA PCR products were purified and used as the template for in vitro transcription (IVT) using the MEGAshortscript T7 kit (Life Technologies, AM1354) and BE4max mRNA were in vitro transcribed using mMESSAGE mMACHINE T7 ULTRA kit (Life Technologies, AM1345). The transcribed products were purified using the MEGA Transcription Clean‐up kit (Life Technologies, AM1908) and eluted in RNase‐free water.^21^ BE4max mRNA (100 ng/ul) and sgRNA (50 ng/ul) were mixed and injected into the cytoplasm of fertilized eggs. Then, the injected zygotes were cultured in egg water. After microinjection for 24 h, the tail tissues were dissected for genotyping. Then, zebrafishes were cultured for 3 months, and were collected for phenotyping, targeted deep sequencing and RNA‐seq analysis.

### 2.10 Western blot analysis

Western blot analysis was performed according to the methods we previously described.^20^ The antibodies used included anti‐PKM2 (1:2,000; Proteintech) and anti‐GAPDH (1:20 000; Proteintech). Images were captured with Tannon 5200SF.

### 2.11 Data accession

Targeted deep sequencing and RNA‐seq data have been deposited into Sequence Reads Archive (SRA: PRJNA716725). The authors declare that all used plasmids, annotated DNA sequences and other related data are all available from the authors upon request.

### 2.12 Statistical analysis

Statistical analysis was performed using GraphPad Prism 8.0. Results were obtained from 2 or 3 independent experiments. Data were presented as the mean ± SD values. Student's *t* test was performed for statistical analysis. *P < .05, **P < .01, ***P < .001 and ****P < .0001.

## 3 RESULTS

### 3.1 Base editing‐mediated efficient mutation of splicing sites of *PKM*

**FIGURE 1:**
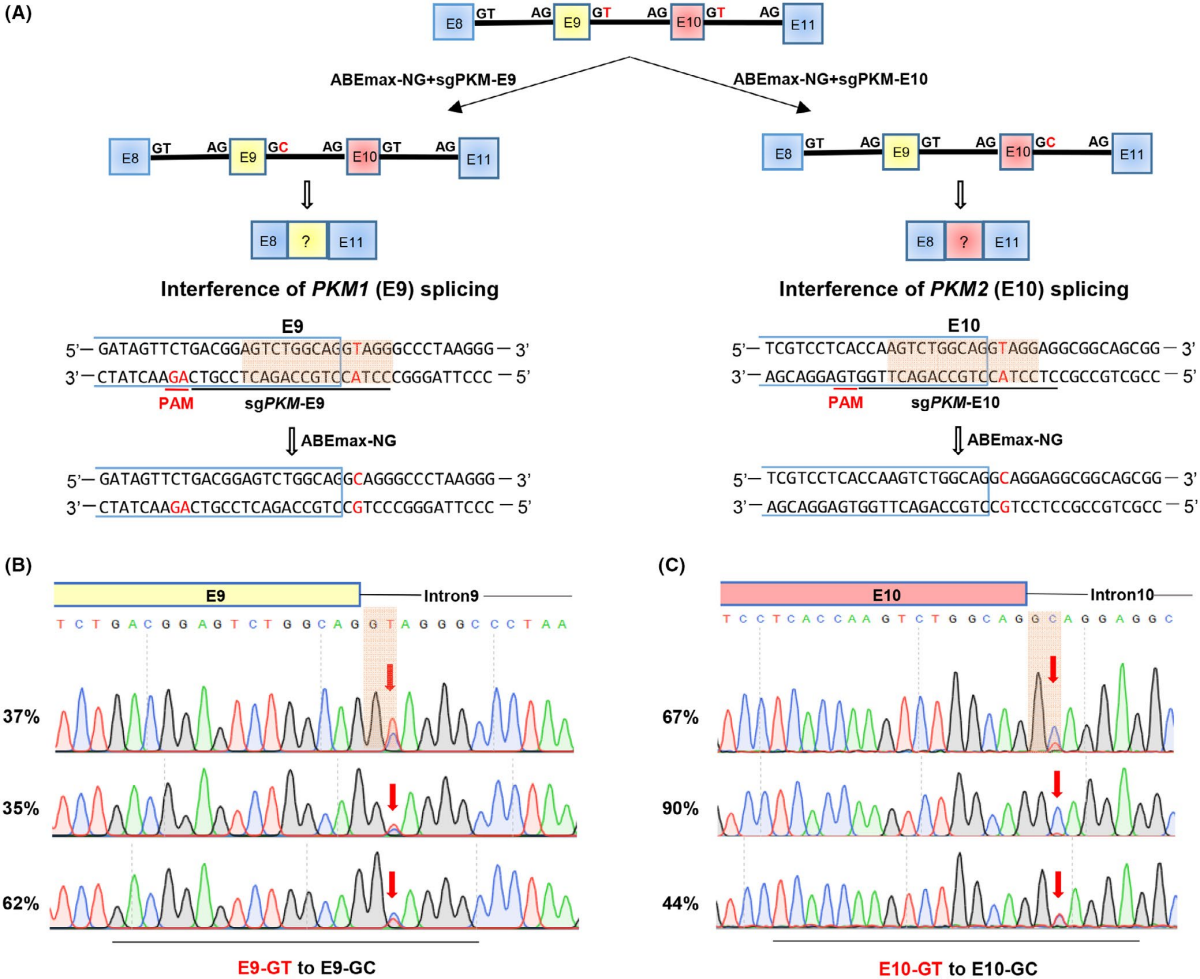
Base editing‐mediated efficient mutation of splicing junction sites of *PKM* gene. A, Schematic of ABEmax‐NG induced T‐to‐C in *PKM* gene. *PKM1* contains exon 9 in yellow square, and *PKM2* contains exon 10 in red square. The gRNA sequence is underlined in black and the PAM sequence is underlined in red. The targeted splicing sites are highlighted in red. B‐C. The mutation efficiencies of ABEmax‐NG‐induced T‐to‐C in E9‐GT B, or E10‐GT C, mutant cells were analysed from Sanger sequencing data using EditR

*PKM1* and *PKM2* that are spliced from pre‐mRNA transcripts exclusively possess exon 9 or 10 respectively (Figure S1A); thus, disruption of the skipping rules of exon 9 or 10 can perturbate splicing process. Previously, the 5’ donor sites of ‘GT’ within the introns 9 and 10 of *PKM* have been successfully mutated into ‘AT’ by CRISPR‐guided AID, while it produces some unwanted base conversions within the scope of gRNA, including exon and intron bases.^17^ To test whether CBEs or ABEs can be used for precise mutation of 5’ donor sites of ‘GT’ within the introns 9 and 10 of *PKM* into ‘GC’ in human cells (Figure 1A), we designed 2 gRNAs with ABEmax‐NG, which preferentially recognizes ‘NGN’ protospacer‐adjacent motif (PAM) sequence (Figure S1B).^18^ With this strategy, only 1 ‘T’ was located within the editing window of ABEmax‐NG.^5^

The transfected HCT116 colon cancer cells with ABEmax‐NG and gRNAs were subjected to Sanger sequencing. As expected, the GT‐to‐GC conversion efficiency targeting intron 9 was 35%‐62% and the efficiency targeting intron 10 was 44%‐90%, without observable unwanted mutations (Figure 1B‐C). To further test the feasibility of targeting the 3’ acceptor sites, we designed another 2 gRNAs to convert ‘AG’ into ‘AA’ of 3’ acceptor sites of introns 9 and 10 of *PKM* with BE4max or BE4max‐NG (Figure S1C). Notably, both 3’ acceptor sites were efficiently mutated by BE4max (intron 9:66%; Figure S1D) and BE4max‐NG (intron 10:93%; Figure S1E), and no apparent mutations were observed in non‐target bases. These results demonstrate that base editors, especially those with minimized editing scope and PAM‐less editors, are suitable for precisely mutating splicing sites (5’ donor sites or 3’ acceptor sites) without by‐products and minimal indels.

### 3.2 Splicing site mutations of *PKM* disrupt endogenous isoform‐specific gene splicing

**FIGURE 2:**
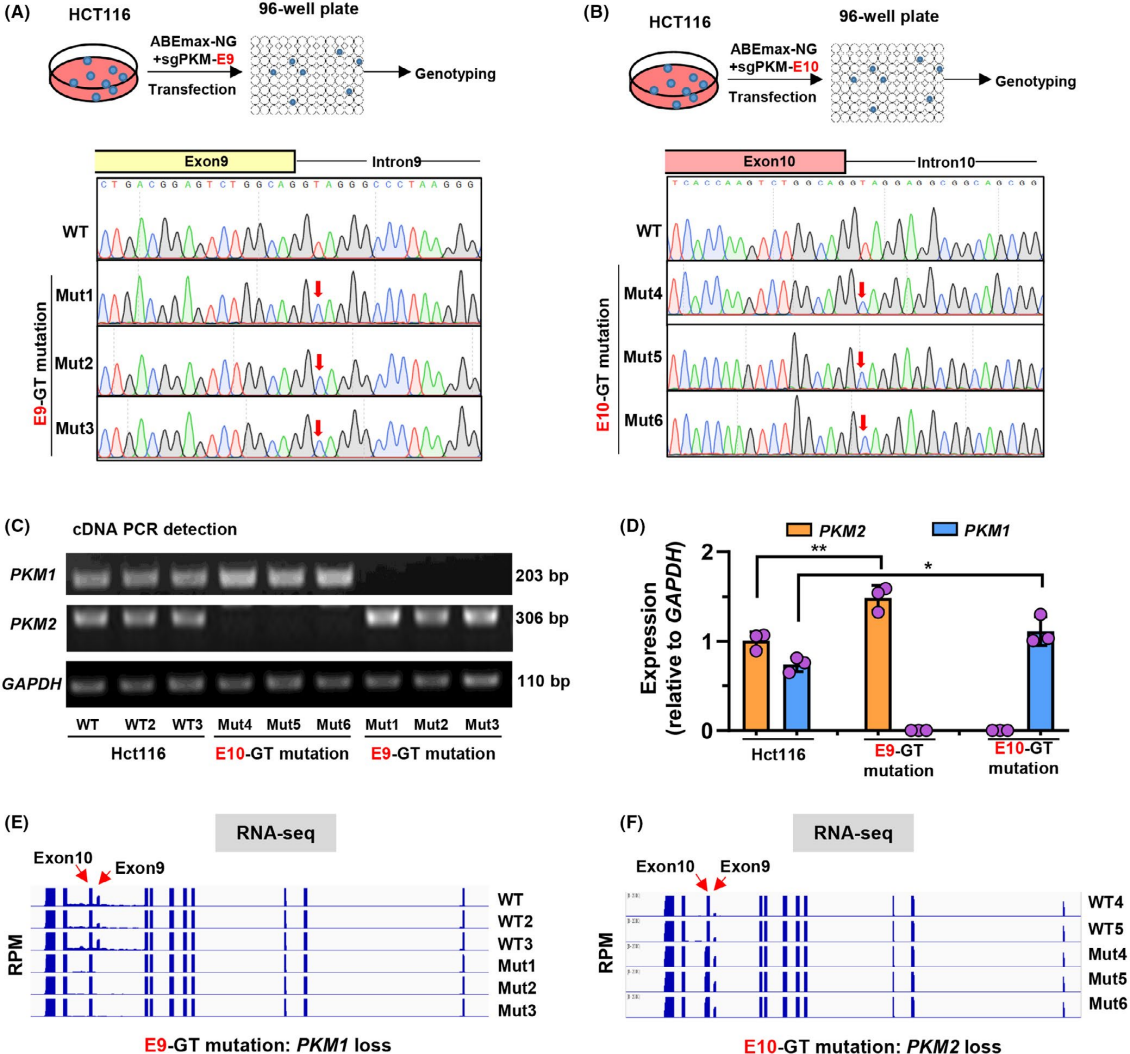
Splicing junction site mutations of *PKM* gene disrupt endogenous isoform‐specific gene splicing. a‐b. Schematic diagram of establishment of E9‐GT A, and E10‐GT B, mutant cell lines from single clones. The E9‐GT mutant cells were diluted and plated onto 96‐well plates for culture. After 10 d, the single clone‐derived cell lines were subjected to Sanger sequencing for genotyping. Three single clone‐derived cell lines were established for E9‐GT (Mut1‐3) and E10‐GT (Mut4‐6) mutant HCT116 cells. C, Detection of *PKM1* and *PKM2* expression by gel electrophoresis with expected length of PCR fragments in wild‐type (WT, WT2 and WT3), E9‐GT (Mut1‐3) and E10‐GT (Mut4‐6) mutant HCT116 cell lines. D, Semi‐quantitative analysis of *PKM1* and *PKM2* expression relative to GAPDH in c using ImageJ software. E‐F. Total RNA from wild‐type (WT, WT2 and WT3), E9‐GT (Mut1‐3) and E10‐GT (Mut4‐6) mutant HCT116 cell lines was subjected to RNA‐seq analysis. The enrichment of transcripts within exons or introns was presented by IGV viewer

Splicing sites have been successfully mutated,^16^, ^17^, ^18^ while the detailed effects of these mutations on RNA splicing have not been explored yet. To analyse this in a clean genetic context, we established single‐clone–derived mutant cell lines from transfected HCT116 cells with GT‐to‐GC mutations within the introns 9 (Mut1‐3; E9‐GT mutation) and 10 (Mut4‐6; E10‐GT mutation) of *PKM*, and all those cell lines were validated by Sanger sequencing with 100% mutation rate (Figure 2A‐B). Meanwhile, 3 single‐clone–derived wild‐type (WT, WT2 and WT3) lines were also established as controls in subsequent analysis.

Wild‐type or mutant HCT116 cells were collected for RNA extraction and reverse transcription (RT). Then *PKM1*‐specific primers spanning exons 9 and 11 and *PKM2*‐specific primers spanning exons 10 and 11 were used for amplifying and detecting *PKM1* and *PKM2* by 1% agarose electrophoresis respectively. Intriguingly, *PKM1* was completely lost in Mut1‐3 cell lines, while *PKM2* was completely lost in Mut4‐6 cell lines (Figure 2C). We also half‐quantified the expression levels of *PKM1* or *PKM2* relative to *GAPDH* and demonstrated that the expression levels of *PKM2* or *PKM1* were increased in Mut1‐3 or Mut4‐6 cell lines respectively (Figure 2C‐D). We also performed RNA‐seq analysis to detect alternative splicing isoforms of *PKM* transcripts. In Mut1‐3 cell lines with E9‐GT mutation, the transcripts spanning exon 9 were diminished (Figure 2E), which was consistent with RT‐PCR results. However, we indeed observed not only the upregulation of exon 9 transcripts in Mut4‐6 cell lines with E10‐GT mutation but also observed the extension of transcripts around exon 10 (Figure 2F), which will be explored subsequently in detail. Collectively, with CRIPSR‐based base editing strategy to mutate splicing sites, we successfully perturbated endogenous *PKM* splicing and established a cell model with specific isoform expression (with endogenous *PKM1* loss or *PKM2* loss).

### 3.3 *PKM1* and *PKM2* differentially regulate cell growth and cell cycle–related gene expression

Because of the lack of appropriate techniques for functional analysis of endogenous *PKM* isoforms, the function of *PKM* remains controversial till now. For instance, *PKM2* is generally considered to promote tumour growth,^24^ whereas it has been demonstrated that *PKM2* is dispensable for tumour growth and development,^25^ possibly due to different research models and ambiguous genetic background. Thus, we decided to investigate isoform‐specific functions with our cell model with endogenous *PKM1* or *PKM2* loss.

**FIGURE 3:**
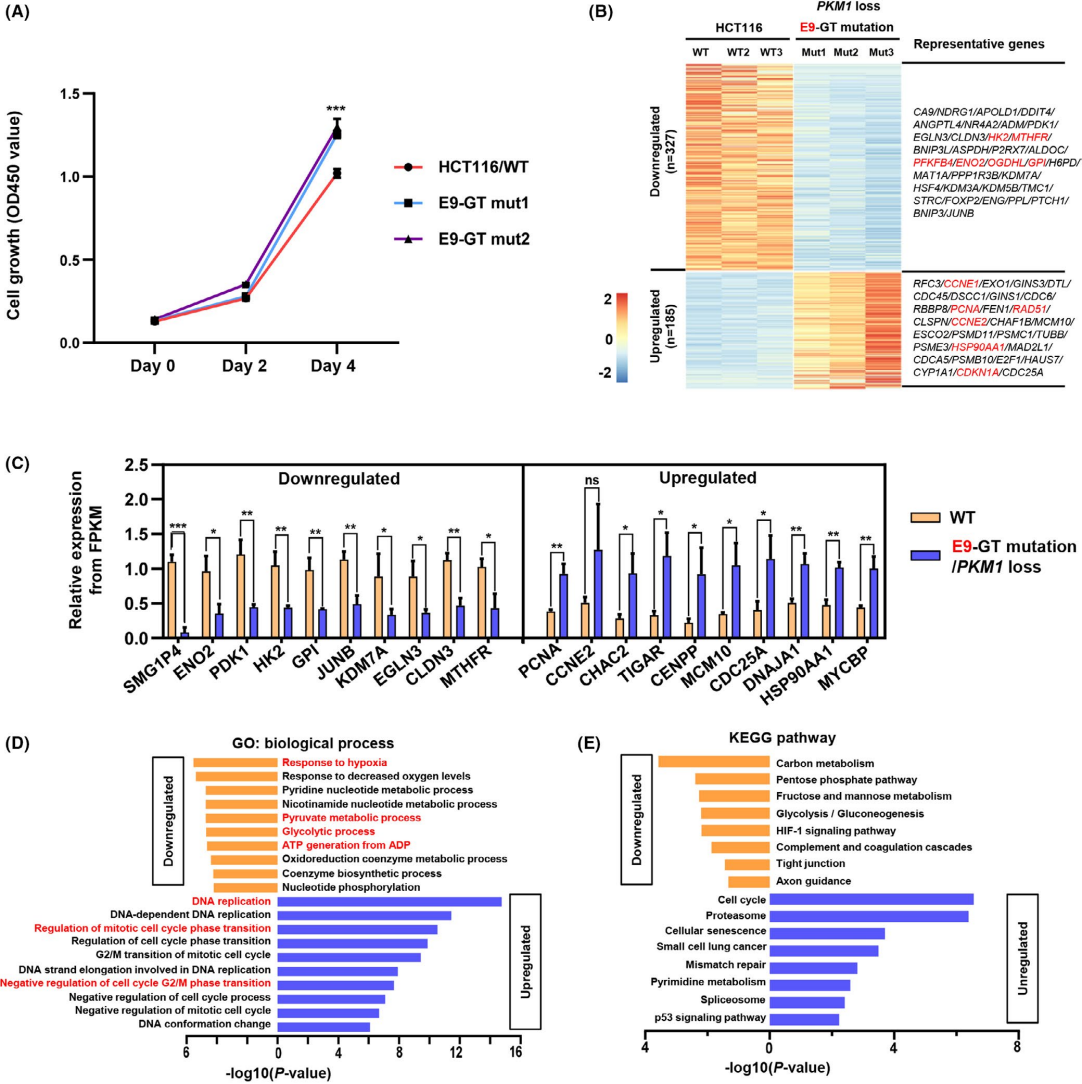
*PKM*/E9‐GT mutation promotes cell growth and activates cell cycle–related gene expression. A, E9‐GT mutation promotes cell growth. CCK‐8 kit was used to analyse the cell growth of single‐clone–derived E9‐GT‐mutant cells and wild‐type HCT116 cells were used as a control. Absorbance was measured at OD450 at 0, 48 and 96 h. B, Wild‐type and E9‐GT‐mutant (*PKM1* loss) cells were subjected to RNA‐seq analysis, and differentially expressed genes (upregulated and downregulated) were presented as a heatmap. Representative differentially expressed genes were also presented. C, The relative expression of differentially expressed genes was analysed from FPKM values in RNA‐seq data. The mean values from 3 single‐clone–derived E9‐GT‐mutant cell lines were shown. D, GO analysis of the differentially expressed genes upon E9‐GT mutation. E, KEGG pathway analysis of the differentially expressed genes upon E9‐GT mutation

**FIGURE 4:**
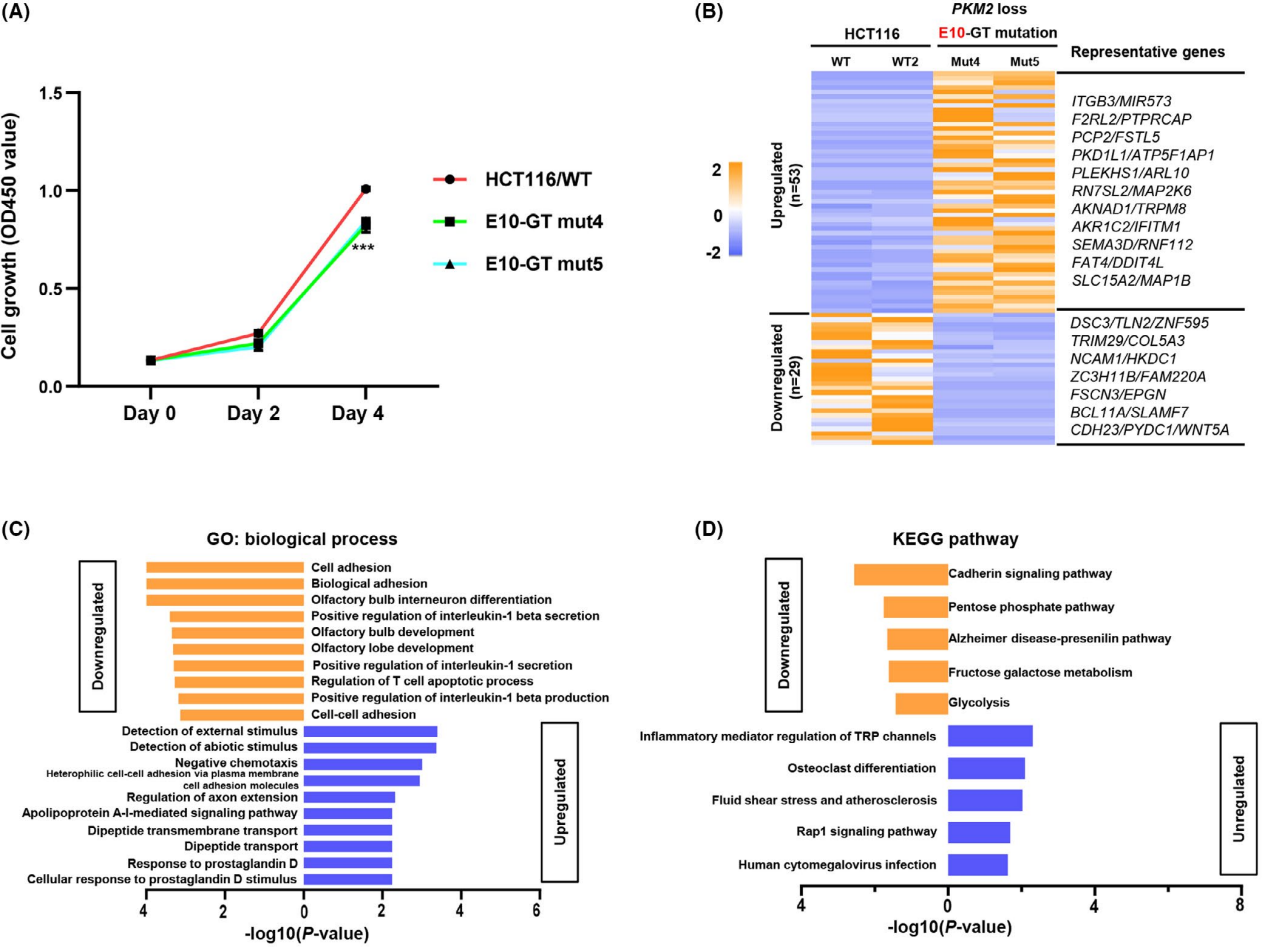
*PKM*/E10‐GT mutation inhibits cell proliferation and related gene expression. A, E10‐GT mutation inhibits cell growth. CCK‐8 kit was used to analyse the cell growth of single‐clone‐derived E10‐GT‐mutant cells and wild‐type HCT116 cells served as a control. Absorbance was measured at OD450 at 0, 48 and 96 h. B, RNA‐seq analysis of differentially expressed genes in E10‐GT‐mutant cells relative to wild‐type HCT116 cells. Representative differentially expressed genes were also presented. c‐d. GO C, and KEGG D, pathway analysis of the differentially expressed genes responding to E10‐GT mutation

Cell growth was determined by using CCK‐8 Cell Counting Kit, showing that *PKM1* loss and *PKM2* increase promoted tumour cell growth in E9‐GT mutation cells (Figure 3A). In contrast, *PKM2* loss and *PKM1* increasement decelerated tumour cell growth in E10‐GT‐mutant cells relative to control HCT116 cells (Figure 4A). Transcriptome analysis demonstrated that the expression of several cell cycle promoters, such as *PCNA*, *CCNE1*, *CDKN1A*, etc, was upregulated upon *PKM1* loss (Figure 3B‐C), and those genes were mainly related to DNA replication, cell cycle phase transition and oncogenic pathways (Figure 3D‐E). Conversely, the expression of multiple genes associated with hypoxia response, pyruvate metabolism and glycolytic process, such as *ENO2*, *HK2*, *GPI*, *MTHFR*, etc, was strongly downregulated in E9‐GT‐mutant cells (Figure 3B‐D). It is worth noticing that the downregulated genes were mainly involved in carbon metabolism, pentose phosphate pathway, glycolysis and HIF‐1 signalling pathway. These transcriptional consequences might be mainly elicited by *PKM2* upregulation, which is consistent with the notion that *PKM2* plays important roles in glycolysis to achieve the nutrient demands of cancer cell proliferation.^26^

On the other hand, there were only 29 downregulated genes and 53 upregulated genes with fold‐change >2 in E10‐GT‐mutant cells with *PKM2* loss (Figure 4B). Downregulated genes, including *HKDC1*, *ZNF595*, *CDH23*, *WNT5A*, etc, were related to cell adhesion, pentose phosphate pathway and glycolysis (Figure 4C). Actually, the expression of cell cycle regulator *PCNA* and *CDKN1A* was also decreased upon E10‐GT mutation. Upregulated genes, including *ITGB3*, *PKD1L1*, *RN7SL2*, *DDIT4L*, etc, were associated with external stimulus, negative chemotaxis and inflammatory response (Figure 4D). Through our transcriptome analysis, we conclude that *PKM1* and *PKM2* may co‐ordinately regulate cell cycle‐ and metabolism‐related genes and signalling pathways in cell growth. In sum, specific loss of *PKM* isoform reveals differential roles in cell cycle regulation and tumour cell growth.

### 3.4 Interference of endogenous *PKM* splicing generates novel splicing isoforms

**FIGURE 5:**
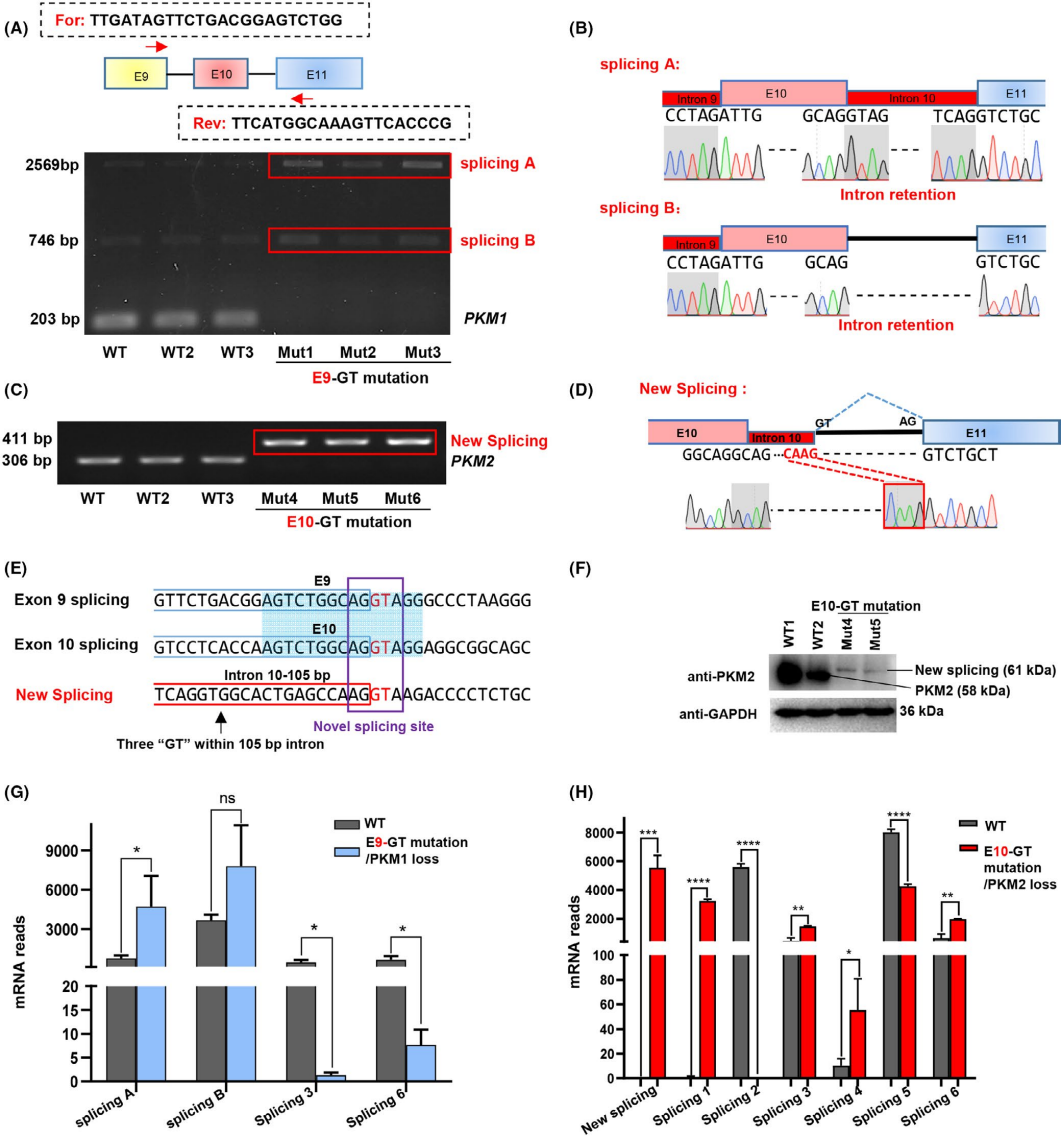
Interference of endogenous *PKM* splicing generates novel splicing isoforms. A, Detection of splicing isoforms in wild‐type HCT116‐ and E9‐GT‐mutant cells. Two isoforms, defined as splicing A and B, were detected in RT‐PCR analysis. B, Analysis of DNA sequences of splicing A and B by Sanger sequencing. The intron retention was presented as red box. DNA sequences spanning the previous exons and introns were also presented. C, Detection of splicing isoforms in wild‐type HCT116 and E10‐GT‐mutant cells. A new splicing isoform was detected in RT‐PCR analysis. D, Analysis of DNA sequences of the new splicing in c by Sanger sequencing. E, Comparison of DNA sequences around the splicing sites neighbouring exons 9 and 10 and the new splicing. The splicing donor sites of ‘GT’ were highlighted in red. F, Western blot analysis of PKM2 and GAPDH expression in wild‐type and E9‐GT‐mutant cells (Mut4 and Mut5 clones). G‐H. Comparison of the mRNA reads of differential splicing isoforms resulted from E9‐GT mutation F, or E10‐GT mutation G, in RNA‐seq analysis

Splice site mutation always disrupts native splicing of target genes in mammalian, such as exon skipping and intron retention,^27^ and causes many kinds of diseases.^28^ In E10‐GT‐mutant cells, we indeed observed intron retention in RNA‐seq tracks (Figure 2F), which was observed as *PKM2* loss in PCR detection (Figure 2C), indicating that the splicing isoforms should be explored in detail. Indeed, except *PKM1* loss in E9‐GT‐mutant cells, we observed the upregulation of 2 splicing isoforms, which have not been identified previously and might be intermediate mRNA products, including a 2,569 bp length cDNA fragments with introns 9 and 10 retention (splicing A) and a 746 bp length cDNA fragments with intron 9 retention (splicing B) (Figure 5A‐B). Meanwhile, we detected a new splicing form accompanied by *PKM2* loss in E10‐GT‐mutant cells in PCR amplification (Figures 2 and 5; in Figure 2D, the new splicing band was not shown). Sanger sequencing demonstrated that 105 bp bases within intron 10 were retained in this new splicing form (Figure 5D). We compared the sequences between the splicing junction sites of exons 9 and 10, and the new splicing form. We found that DNA sequences neighbouring the donor sites of ‘GT’ for introns 9 and 10 were highly conservative, and the 3’‐end sequences of the 105 bp new exon were ‘AG’, which was consistent with exons 9 and 10 (Figure 5E). There were 3 ‘GT’ within this 105 bp new exon, while only the 4th ‘GT’ was 5’ neighbouring with ‘AG’ and designated as the splicing donor site, which might be the key triggering signal for producing a new splicing form. We further found that the protein level of the new splicing isoform was much lower than wild‐type PKM2 protein (Figure 5F), suggesting that the new splicing protein resulted from intron retention might be less functional.

To systematically compare the splicing events upon E9‐GT or E10‐GT mutation, we analysed all splicing transcripts in RNA‐seq data. In total, we identified another 6 novel splicing isoforms (splicing 1‐6) with various intron retention, as well as exon skipping in splicing 3, as presented in Figure S2A‐B. In E9‐GT‐mutant cells, mRNA reads of splicing 3 and 6 were decreased (Figure 5G); in E10‐GT‐mutant cells, splicing 3, 4, and 6 were increased, splicing 1 was newly generated, splicing 5 was decreased, and splicing 2 with intron 10 retention was completely lost (Figure 5H). Relative to classical *PKM* isoforms (*PKM1* and *PKM2*), the mRNA reads of those newly identified or produced splicing forms were much lower, and the downregulation of some isoforms might be elicited by the nonsense‐mediated decay effects with the appearance of premature termination codons in extended transcripts within introns 8 and/or 10 (Figure S2A‐B), while it may generate undesirable or uncontemplated consequences. These data remind us to check the alterations of both canonical and non‐canonical transcripts upon perturbation of splicing events with base editing strategy‐mediated mutation of splicing sites.

### 3.5 Base editing‐mediated splicing site mutation in vivo

**FIGURE 6:**
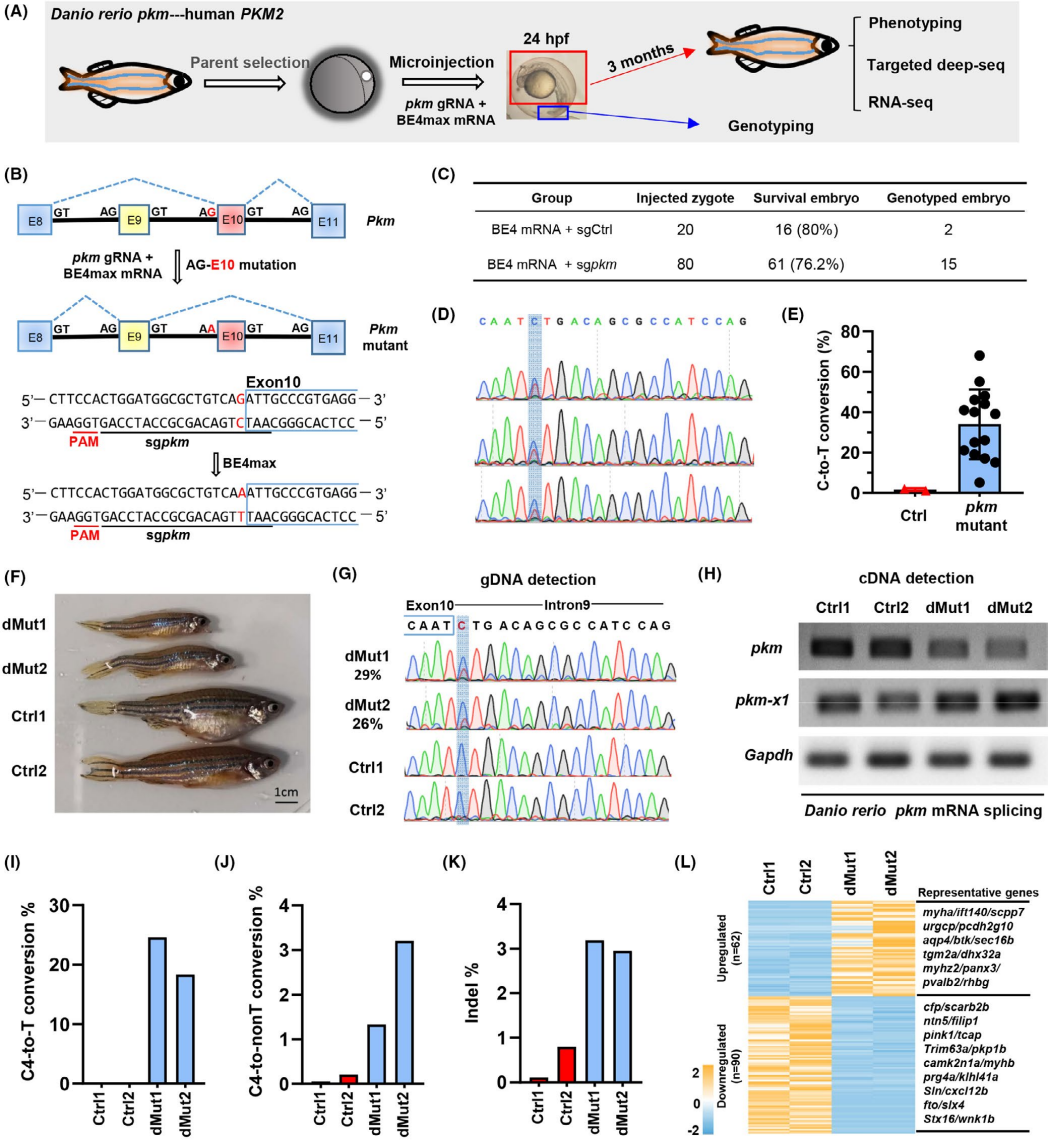
Interference of *PKM* mRNA splicing in zebrafish. A, Schematic diagram of base editing in zebrafish. *pkm* gRNA and BE4max mRNA were microinjected into the zygote. The tail was collected for genotyping after culture for 24 h. Adult zebrafishes were subjected to phenotyping, targeted deep sequencing (deep‐seq) and RNA‐seq analysis after culture for 3 months. B, Schematic diagram of BE4max‐induced C‐to‐T in *pkm* of zebrafish. *pkm* contains exon 10 and *pkm‐x1* contains exon 9. The gRNA sequence targeting AG‐exon 10 is underlined in black and the PAM sequence is underlined in red. The targeted G‐to‐A conversion is highlighted in red. C, Base editing in zebrafish. BE4max mRNA and gRNA targeting GFP (Ctrl) or *pkm* were co‐injected into zygotes. The number of survival embryos and survival rates were calculated. Moreover, the number of embryos with genotyping results was also provided. D, The chromatogram of Sanger sequencing showing examples of BE4max‐induced C‐to‐T conversions in zebrafish embryos. E, Statistical analysis of the C‐to‐T conversion frequencies induced by BE4max using EidtR in zebrafish embryos. Ctrl, control gRNA group. F, The morphological analysis of control (Ctrl1 and Ctrl2) and mutant (dMut1 and dMut2) zebrafishes. G, The chromatogram of Sanger sequencing for zebrafishes in F. H, RT‐PCR detection of *pkm* and *pkm‐x1* from control (Ctrl1 and Ctrl2) and mutant (dMut1 and dMut2) zebrafishes. PCR fragments were analysed by agarose electrophoresis. I‐K, Targeted deep sequencing analysis of genomic DNA fragments containing the targeting site. The on‐targeting C4‐to‐T conversion rates I, C4‐to‐non‐T conversion rates J, and indel rates were presented in control (Ctrl1 and Ctrl2) and mutant (dMut1 and dMut2) zebrafishes. l. Clustering analysis of mutation zebrafish and normal fish. RNA‐seq analysis of differentially expressed genes in control (Ctrl1 and Ctrl2) and mutant (dMut1 and dMut2) zebrafishes

To perturbate endogenous splicing of *PKM* in vivo, we choose zebrafish (*Danio rerio*) as our research model to mutate splicing sites, in which the coding sequence of *pkm* is quite similar with human *PKM2*.^29^ After analysing the sequence features of the donor sites and acceptor sites on introns 9 and 10, we designed a gRNA targeting the acceptor site of ‘AG’ within intron 9 with BE4max to induce AG‐to‐AA mutation in zebrafish. Briefly, we selected proper zebrafish parents for genotyping, and established a zebrafish line with homozygous *PKM* coding DNA sequences for base editing in vivo. In vitro transcribed BE4max and gRNA were transcribed and micro‐injected into the zygotes. Zebrafishes were collected at embryonic or adult stages for genotyping and phenotyping analyses (Figure 6A).

To test the targeting efficiency of BE4max in zebrafish embryo, we co‐injected BE4max mRNA and a gRNA targeting *tyr* into zygotes, which were expected to induce P302S mutation,^30^ and embryos were collected for genotyping at 24 h post‐injection (Figure S3A). Among seven collected embryos, four embryos were highly edited with expected C‐to‐T conversions, and mutation rate was as high as 76% (Figure S3B‐C). These data validate the good feasibility of BE4max for precise base conversions in zebrafish.

Then, we started to generate mutant zebrafish using BE4max and a gRNA targeting the acceptor site of ‘AG’ within intron 9, which was expected to be able to induce only 1 ‘G‐to‐A’ conversion (AG‐E10 mutation) within the editing scope of BE4max (Figure 6B). We obtained 61 survival embryos from 80 injected zygotes, and the survival rate of *pkm*‐targeted embryos (76.2%) was close to the control group (80%) targeting GFP (Figure 6C), indicating that the cellular toxicity of BE4max system is low and acceptable for gene manipulation in zebrafish embryo. Among 15 edited embryos with genotyping results, all embryos were successfully edited (Figure 6D) with editing efficiency ranging 5%‐68%, with expected G‐to‐A’ conversions (‘C‐to‐T’ at the complementary strand) (Figure 6E). Notably, splicing site mutant zebrafishes with 29% (dMut1) and 26% (dMut2) editing efficiencies were much smaller than control zebrafishes (Figure 6F‐G). To further prove that the phenotype was elicited by the alterations of *pkm* splicing upon AG‐E10 mutation, the expression of *pkm‐x1* and *pkm*‐mutant in wild‐type and AG‐E10‐mutant zebrafishes was detected by RT‐PCR. The primers for amplifying *pkm*‐mutant were designed referring to human *PKM2* (Figure S3D); therefore, *pkm‐x1* and *pkm*‐mutant were expected to be human *PKM1* and *PKM2* analogues respectively. It demonstrated that the expression of *pkm*‐mutant was decreased while the expression of *pkm‐x1* was increased upon AG‐E10 mutation (Figure 6H). Thus, we speculate that consistent with the findings with decreased cell growth in E10‐GT‐mutant HCT116 cells, the embryo growth retardation might be elicited by *pkm*‐mutant (*PKM2* analogue) decrease and *pkm‐x1* increase. This observation is also in line with the notion that *PKM2* highly expressed in human embryos is essential for embryo development.^31^ Previously, base editing strategy has been applied to introduce splicing site mutations in mouse embryos, while no developmental or disease‐related phenotypes were observed.^18^ In the present study, we observed phenotypic defects for edited zebrafish embryos within splicing site mutations.

Next, we further detected the proportion of by‐products along with expected C‐to‐T conversions within the acceptor site of ‘AG’ in intron 9 by targeted deep sequencing. The on‐targeting efficiency from the deep sequencing data was generally consistent with Sanger sequencing results (Figure 6I). We also found 1.3% and 3.2% of C‐to‐non‐T conversions (Figure 6J) as well as 3.1% and 2.9% of indels (Figure 6K) in the mutant zebrafishes (dMut1 and dMut2). We also performed RNA‐seq analysis to detect the global transcriptional alterations upon AG‐E10 mutation. In total, there were 62 upregulated genes (ie, *myha*, *scpp7*, *tgm2a*, etc) and 90 downregulated genes (ie, *cfp*, *scarb2b*, *filip1*, *wnk1b*, etc) (Figure 6L). Gene ontology (GO) analysis demonstrated that the downregulated genes were mainly related to mesenchyme morphogenesis, protein refolding and glutamate receptor signalling pathway, which were related to mesodermal development and development‐related processes (Figure S4A). KEGG pathway analysis showed that the downregulated genes were mainly involved in 2‐oxocarboxylic acid metabolism and amino acid biosynthesis and metabolism (Figure S4B). It suggests that *pkm* promotes embryo development through modulating 2‐Oxocarboxylic acid and amino acid metabolism in zebrafish. Thus, we validate the perturbation of splicing in vivo through base editing‐mediated splicing site mutations, and we also established an in vivo model to study isoform‐specific functions in embryonic development.

## 4 DISCUSSION

**FIGURE 7:**
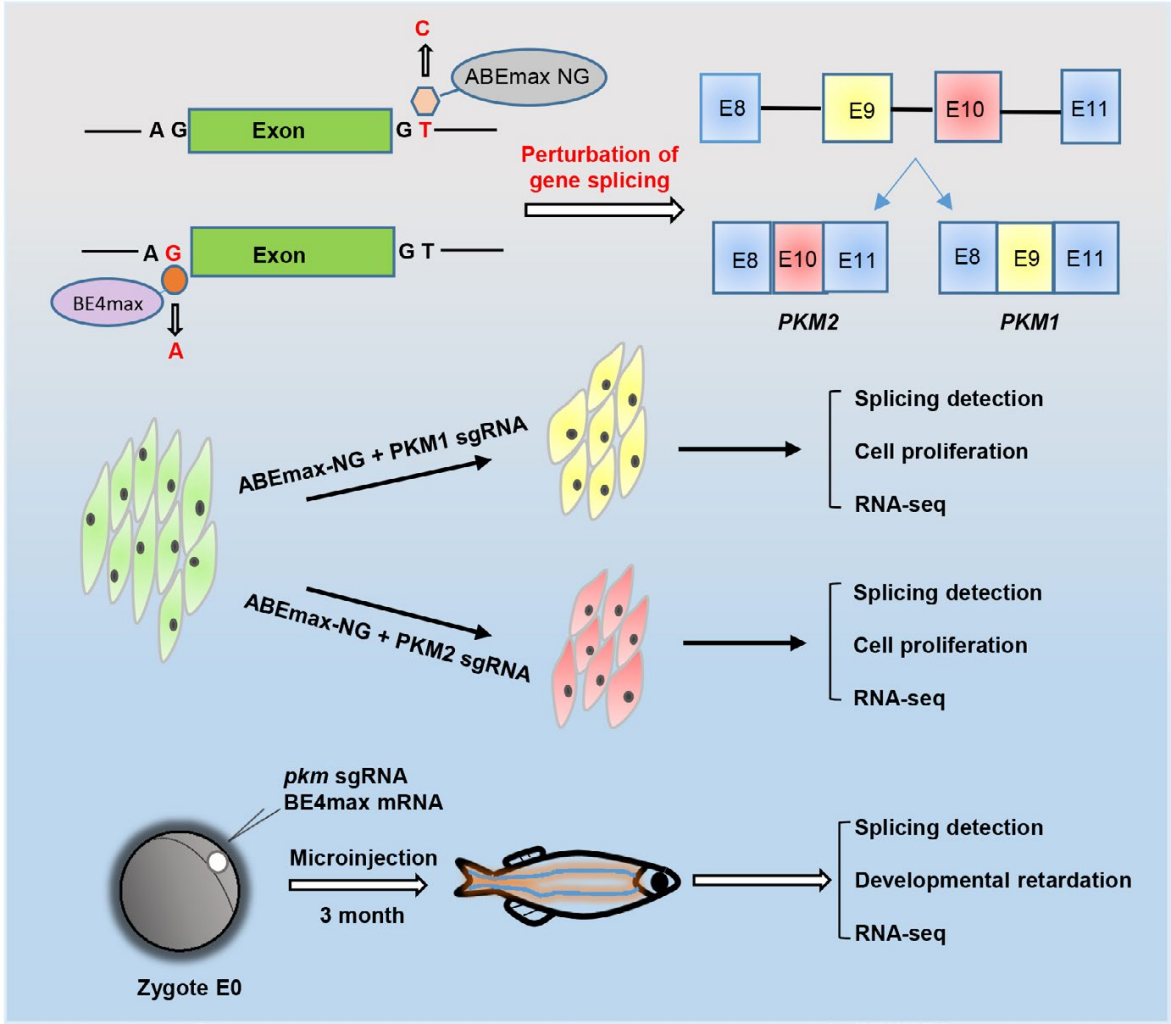
Model for base editing‐mediated splicing perturbation in functional analysis. Because of the lack of appropriate techniques, the specific functions of the 2 *PKM* splicing isoforms have not been clarified endogenously yet. In this study, we used CRISPR‐based base editors to perturbate the endogenous alternative splicing of *PKM* by introducing mutations into the splicing junction sites in HCT116 cells and zebrafish embryos. It is proved that CRISPR‐based base editing strategy can be used to disrupt the endogenous alternative splicing of genes of interest to study the function of specific splicing isoforms in vitro and in vivo

The human diversification transcriptome largely relies on vast RNA splicing, and RNA mis‐splicing underlies a growing number of human diseases.^28^ Thus, elucidation of isoform‐specific functions in an endogenous status is of great significance. Here, we utilized CRISPR‐based base editing strategy and successfully mutated the donor sites and acceptor sites of introns within *PKM* coding sequences in vitro and in vivo (Figure 7), which resulted in the perturbation of endogenous isoform splicing, alterations of cell proliferation rates and related gene expression (Figures 3 and 4), as well as developmental defects in zebrafish (Figure 6). It further validates the key roles of alternative *PKM* splicing events required for tumour cell proliferation^32^ and normal embryo development.^31^

In single‐clone–derived mutant HCT116 cells, native *PKM1* or *PKM2,* were completely lost upon E9‐GT or E10‐GT mutation respectively (Figure 2). It provides a good cellular model for loss‐of‐function analysis of isoform‐specific functions, which is not feasible for RNA interference or CRISPR‐mediated gene knockout. Based on this finding, we conclude that for most genes following canonical splicing rules, the splicing donor sites‐GT and acceptor sites‐AG within introns are extremely important for guaranteeing normal splicing events. When splicing sites are mutated, the splicing isoforms will be altered with almost no suspense. In addition, the obtained zebrafishes are mosaic using base editing tools, and it will be helpful to further study PKM‐specific gene functions in vivo if next‐generation zebrafishes with homogenous phenotypes can be obtained.

In addition to *PKM1* or *PKM2* loss upon splicing site mutations, we also found the perturbation of other 8 splicing isoforms, which were newly identified here but already existed in HCT116 cells with relatively low expression (Figure 5). Intriguingly, we observed a novel splicing form with partial intron 10 retention because of the recognition of a novel splicing donor site (Figure 5E). It reminds us to pay more attention to the splicing site mutations or single nucleotide polymorphisms (SNPs) as well as the generated ‘GT’ and ‘AG’ mutations that may be recognized as novel splicing sites, such as G608G within *LMNA* identified in Hutchinson‐Gilford progeria syndrome.^33^ Besides, the perturbation of *PKM1* and *PKM2* expression as well as endogenous splicing forms and novel isoforms, although with low expression levels, makes it complicated than we suspect to draw a solid conclusion for *PKM1‐* or *PKM2‐*specific functions. Therefore, it needs detection of the alternations of all splicing isoforms at transcriptome levels when using base editing strategy to perturbate splicing events. Even so, base editor‐mediated mutation or correction of splicing sites is applying for targeted therapy of mis‐splicing‐associated diseases in animal models.^34^, ^35^, ^36^

In summary, base editing was successfully applied to perturbate endogenous *PKM* splicing in cultured tumour cells and zebrafish model, and *PKM1‐* or *PKM2‐*specific functions and modulated genes were generally described. We also revealed some unexpected splicing consequences upon splicing site mutations, which provide us some scientific findings for understanding splicing rules and diseases‐associated abnormal splicing events as well as technical cautions for gene therapy‐directed splicing perturbations.

## CONFLICTS OF INTEREST

No potential conflicts of interest were disclosed.

## AUTHOR CONTRIBUTIONS

Y. Q. and GP designed, conceived and supervised the work. JL (Jianxiang Lin), SW and QS performed the experiments and data analysis. YQ and JL (Jianxiang Lin) co‐wrote the manuscript. JL (Jie Liu) helped with the experiments and data analysis. SH performed computational analysis.

## DATA AVAILABILITY STATEMENT

The data that support the findings of this study are available from the corresponding author upon reasonable request.

## Supporting information

Fig S1

Fig S2

Fig S3

Fig S4

Table S1‐4
