# Supplementary figures and images for: Base editing‐mediated perturbation of endogenous PKM1/2 splicing facilitates isoform‐specific functional analysis in vitro and in vivo
Source: Cell Prolif. 2021 Jul 9;54(8):e13096. doi: 10.1111/cpr.13096 (PMC8349652; doi:10.1111/cpr.13096)

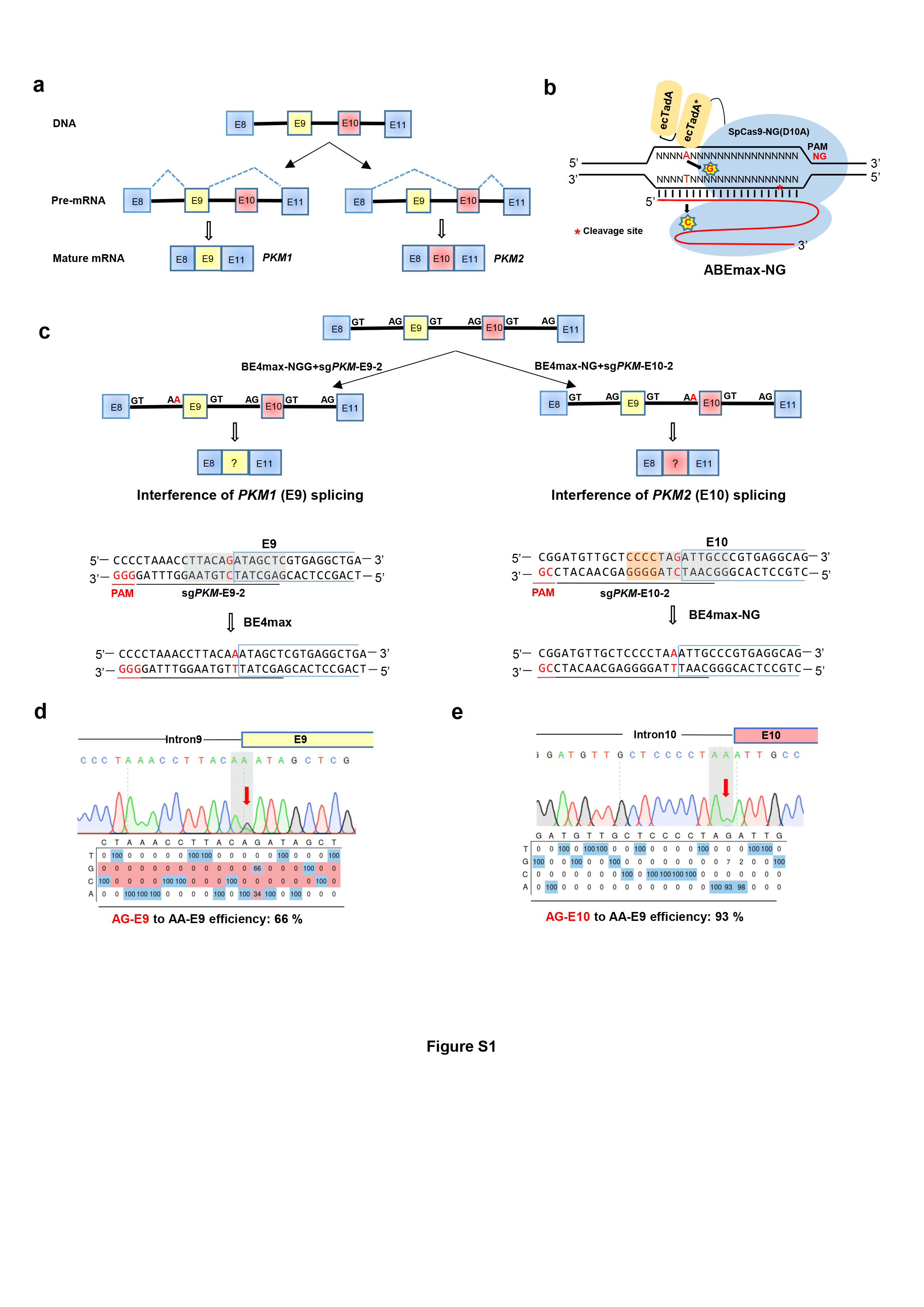

Supplement: Supplementary file 1 — Fig S1 [file CPR-54-e13096-s001.jpg]

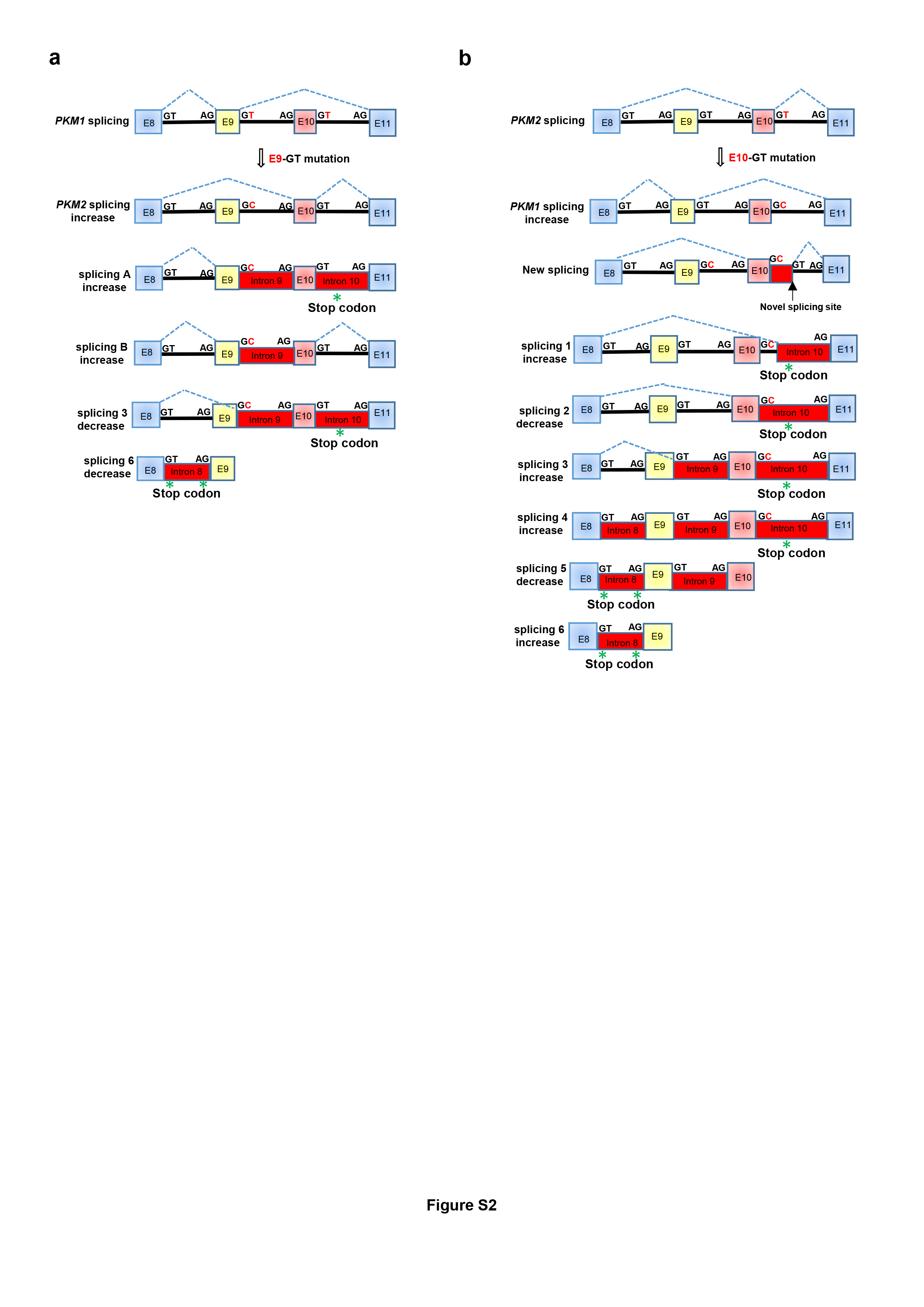

Supplement: Supplementary file 2 — Fig S2 [file CPR-54-e13096-s005.jpg]

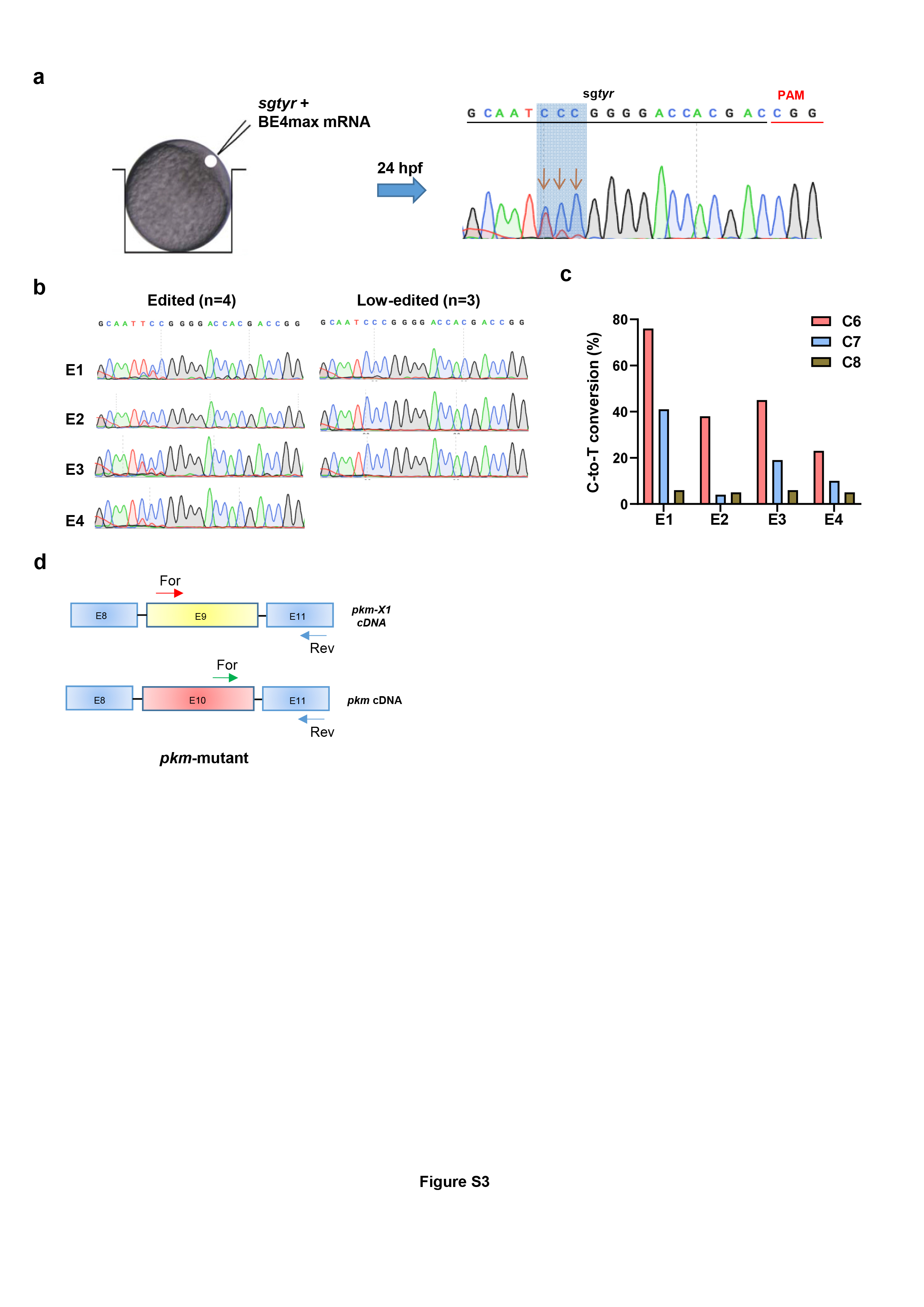

Supplement: Supplementary file 3 — Fig S3 [file CPR-54-e13096-s004.jpg]

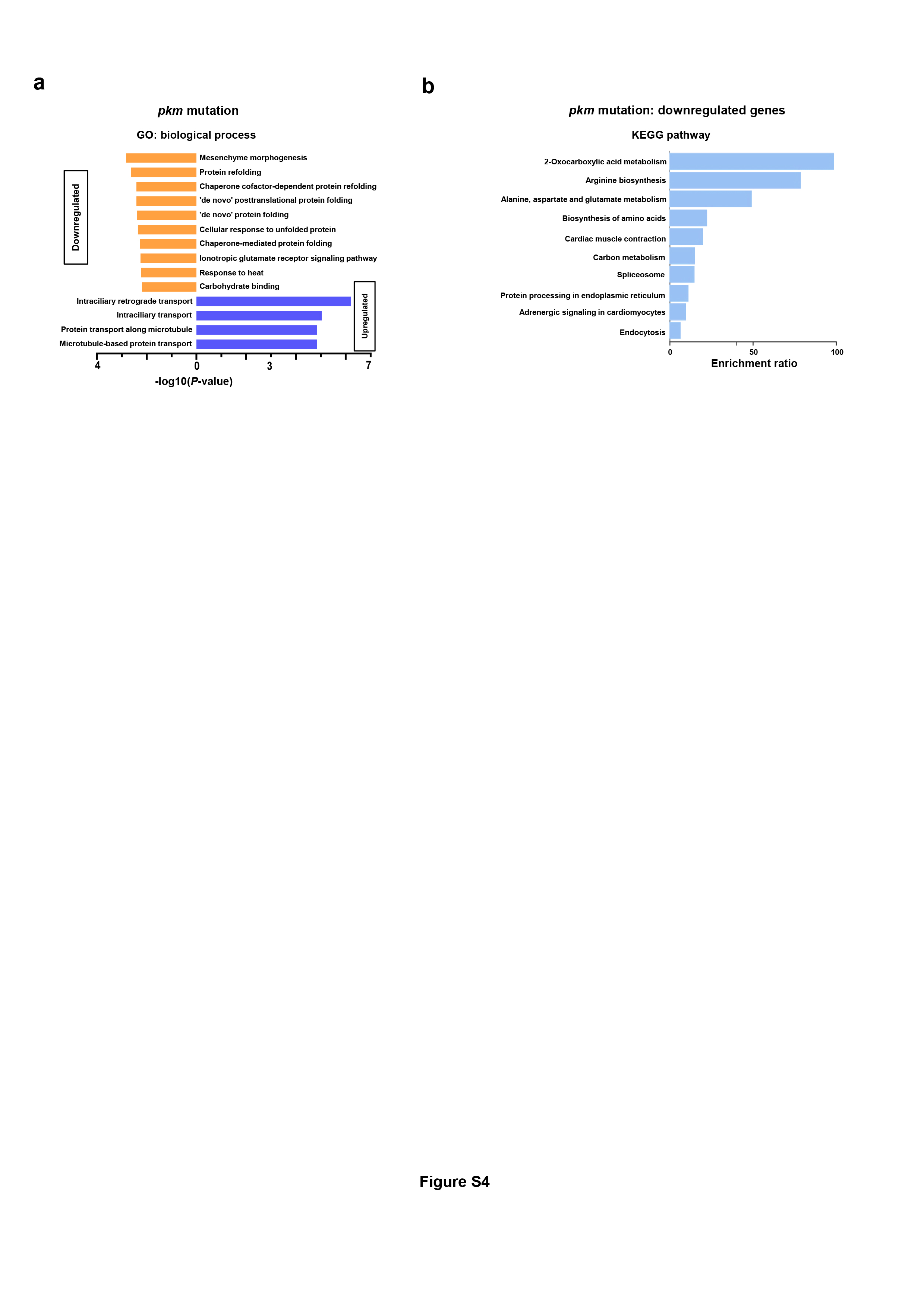

Supplement: Supplementary file 4 — Fig S4 [file CPR-54-e13096-s002.jpg]
