# Supplementary material for: Base editing‐mediated perturbation of endogenous PKM1/2 splicing facilitates isoform‐specific functional analysis in vitro and in vivo
Source: Cell Prolif. 2021 Jul 9;54(8):e13096. doi: 10.1111/cpr.13096 (PMC8349652; doi:10.1111/cpr.13096)
Supplement: Supplementary file 5 — Table S1‐4 [file CPR-54-e13096-s003.docx]

**Supplemental Table 1. Synthesized DNA oligo sequences for sgRNA construction.**

| **Primer Name** | **Primer sequence(5’ to 3’)** |
| --- | --- |
| human PKM-E9-AG-sgRNA-Forward | ACCGagcatctgggaggggacag |
| human PKM-E9-AG-sgRNA-Reverse | AAACctgtcccctcccagatgct |
| human PKM-E9-GT-sgRNA-Forward | ACCGaactcaccaggtgctgcatg |
| human PKM-E9-GT-sgRNA-Reverse | AAACcatgcagcacctggtgagtt |
| human PKM-E10-AG-sgRNA-Forward | ACCGagctatctgtaaggtttag |
| human PKM-E10-AG-sgRNA-Reverse | AAACctaaaccttacagatagct |
| human PKM-E10-GT-sgRNA-Forward | ACCGccctacctgccagactccgtc |
| human PKM-E10-GT-sgRNA-Reverse | AAACgacggagtctggcaggtaggg |
| Danio PKM-sgRNA-Forward | ACCGcaatctgacagcgccatccag |
| Danio PKM-sgRNA-Reverse | AAACtcggatggcgctgtcagattc |
| Ctrl sgRNA-Forward | ACCGtgagaccgagagagggtctca |
| Ctrl sgRNA-Reverse | AAACtgagaccctctctcggtctca |

**Supplemental Table 2. PCR primer sequences for genotyping.**

| **Primer Name** | **Primer sequence(5’ to 3’)** |
| --- | --- |
| human PKM-E9-AG genotyping PCR-Forward | gttaccttcctctctgata |
| human PKM-E9-AG genotyping PCR-Reverse | atctactgtgcctactgag |
| human PKM-E9-GT genotyping PCR-Forward | gttaccttcctctctgata |
| human PKM-E9-GT genotyping PCR-Reverse | atctactgtgcctactgag |
| human PKM-E10-AG genotyping PCR-Forward | ttcccaggaacatgttcctca |
| human PKM-E10-AG genotyping PCR-Reverse | tgagtgctacctagagtcctt |
| human PKM-E10-GT genotyping PCR-Forward | ttcccaggaacatgttcctca |
| human PKM-E10-GT genotyping PCR-Reverse | tgagtgctacctagagtcctt |
| Danio PKM-sgRNA genotyping PCR-Forward | tctgaaggatcctgtaactg |
| Danio PKM-sgRNA genotyping PCR-Reverse | ccaccacatcgcattggctc |

**Supplemental Table 3. PCR primer sequences for RT-PCR.**

| **Primer Name** | **Primer sequence(5’ to 3’)** |
| --- | --- |
| human PKM-GAPDH-For | catgagaagtatgacaacagcct |
| human PKM-GAPDH-Rev | agtccttccacgataccaaagt |
| human PKM1-For | ttgatagttctgacggagtctgg |
| human PKM1-Rev | ttcatggcaaagttcacccg |
| human PKM2-For | attatttgaggaactccgcc |
| human PKM2-Rev | ttcatggcaaagttcacccg |
| Danio GAPDH-For | tgttgtggagtctactggtgt |
| Danio GAPDH-Rev | ggttgacacccatgacaaaca |
| Danio PKMX1-For | cagaggctgccatgtttcatc |
| Danio PKMX1-Rev | gacatccatggcgaagttg |
| Danio PKM-For | cagcggcatcatcatactca |
| Danio PKM-Rev | gacatccatggcgaagttg |

**Supplemental Table 4. PCR primer sequences for targeted deep sequencing.**

| **Primer Name** | **Primer sequences (5’ to 3’)** |
| --- | --- |
| Danio NGS Ctrl1 For | ATCACGggactagtgtatgcattcatg |
| Danio NGS Ctrl1 Rev | CGATGTTttggtgagtatgatgatgcc |
| Danio NGS Ctrl2 For | TTAGGCAGggactagtgtatgcattcatg |
| Danio NGS Ctrl2 Rev | TGACCAGttggtgagtatgatgatgcc |
| Danio NGS Mut1 For | ACAGTGCTggactagtgtatgcattcatg |
| Danio NGS Mut1 Rev | GCCAATttggtgagtatgatgatgcc |
| Danio NGS Mut2 For | CAGATCTggactagtgtatgcattcatg |
| Danio NGS Mut2 Rev | ACTTGAAAttggtgagtatgatgatgcc |
| Danio NGS Mut3 For | GGCTACggactagtgtatgcattcatg |
| Danio NGS Mut3 Rev | CTTGTAttggtgagtatgatgatgcc |
